# Epidemiology of *Helicobacter pylori* infection in dyspeptic Ghanaian patients

**DOI:** 10.11604/pamj.2015.20.178.5024

**Published:** 2015-02-26

**Authors:** Timothy Nii Akushe Archampong, Richard Harry Asmah, Edwin Kwame Wiredu, Richard Kwasi Gyasi, Kofi Nyaako Nkrumah, Kumar Rajakumar

**Affiliations:** 1 Department of Medicine, University of Ghana Medical School, College of Health Sciences, University of Ghana, Legon Boundary, Accra, Ghana; 2 Department of Medical Laboratory Sciences, School of Allied Health Sciences, College of Health Sciences, University of Ghana, Legon Boundary, Accra, Ghana; 3 Department of Pathology, University of Ghana Medical School, College of Health Sciences, University of Ghana, Legon Boundary, Accra, Ghana; 4 Department of Infection, Immunity and Inflammation, University of Leicester Medical School, Leicester LE1 9HN, United Kingdom; 5 Department of Clinical Microbiology, University Hospitals of Leicester NHS Trust, Leicester LE1 5WW, United Kingdom

**Keywords:** Helicobacter, dyspepsia, epidemiology, Ghana, endoscopy, farming, social class

## Abstract

**Introduction:**

*Helicobacter pylori* is a gram-negative urease-producing bacterium causally linked with gastritis, peptic ulcer disease and gastric adenocarcinoma. Infection is more frequent and acquired at an earlier age in developing countries compared to European populations. The incidence of *Helicobacter pylori* infection in dyspeptic Ghanaian patients was 75.4%. However, epidemiological factors associated with infection vary across populations.

**Methods:**

This study used a cross-sectional design to consecutively sample dyspeptic patients at the Endoscopy Unit of the Korle-Bu Teaching Hospital, Accra between 2010 and 2012. The study questionnaire elicited their epidemiological clinical characteristics. *Helicobacter pylori* infection was confirmed by rapid-urease examination of antral biopsies at upper Gastro-intestinal endoscopy.

**Results:**

The sample population of dyspeptic patients attending the Endoscopy Unit for upper GI endoscopy yielded 242 patients of which 47.5% were females. The age distribution of *H. pylori*-infection was even across most age – groups, ranging from 69.2% (61 – 70) years to 80% (21 – 30) years. *Helicobacter pylori* prevalence decreased across areas mapping to the three residential classes in accordance with increasing affluence with rural areas having the highest prevalence. The unemployed and patients in farming had relatively high *Helicobacter pylori* infection rates of 92.3% and 91.7% respectively.

**Conclusion:**

*Helicobacter pylori* is endemic in Ghana but the persistently high prevalence across age groups despite significant community anti-microbial use suggests likely re-crudescence or re-infection from multiple sources in a developing country. Socio-cultural factors such as residential class and farming may be facilitating factors for its continued prevalence.

## Introduction

*Helicobacter pylori* is a spiral-shaped gram-negative urease-producing bacterium [1]. It is the most common chronic bacterial infection known to humans [2, 3]. It is found in the gastric antrum and in areas of gastric metaplasia in the duodenum [4]. It has also been established as the main aetiological agent in the development of chronic gastritis, gastric and duodenal ulceration, gastric B-cell lymphoma and distal gastric cancer [4]. *H. Pylori* has been demonstrated worldwide and in individuals of all ages with conservative estimates suggesting 50% of the world′s population is affected [3]. Infection is more frequent and acquired at an earlier age in developing countries compared to European populations where evidence of *H. Pylori* is rarely found before age 10 but increases to 10% in those between 18 and 30 years of age and to 50% in those older than age 60 [3]. In many developing countries, the infection has a high prevalence rate (80 -- 95%) [5]. More than 50% of children are infected by the age of 10 years with the prevalence of infection rising to over 80% in young adults [6]. The incidence of *H. pylori* infection in Ghanaian patients with dyspeptic symptoms referred for upper gastrointestinal endoscopy at the Korle-Bu Teaching Hospital (KBTH) has previously been found to be 75.4% [7]. The exact mode of transmission is unclear but intra-familial clustering suggests person-to-person spread mainly in childhood [4]. The risk of infection with *H. Pylori* is related to socioeconomic status and living conditions in early life. Overcrowded conditions associated with childhood poverty lead to increased transmission and higher prevalence rates [8]. Potential dietary associations with *H. pylori* have been investigated in humans and chronic excessive salt intake has been shown to enhance *H. pylori* colonization in mice and in humans [9]. This study investigated the epidemiological characteristics of *H. pylori* infection in a West African country with a known high prevalence of the condition.

## Methods

Ethical approval was granted by the Protocol and Ethical Review Committee of the University of Ghana Medical School, College of Health Sciences, Accra, Ghana. This study was conducted in accordance with the Helsinki Declaration. It used a cross-sectional design to consecutively sample dyspeptic patients at the Endoscopy Unit of the KBTH, Accra between April, 2010 and August, 2012. Korle-Bu Teaching Hospital has approximately 2,500 beds and is the main tertiary referral center in the capital, Accra serving the majority of the southern half of Ghana. The Endoscopy Unit runs two four-hour sessions per day on four days of the week. Approximately five patients attend each endoscopy session for upper gastro-intestinal (GI) endoscopy. Sampling was conducted during three such sessions per week. All patients were taken through the explanatory statement of the project before endoscopy. Patients with prior *H. pylori* eradication treatment or proton-pump inhibitor-use two weeks preceding endoscopic analysis were excluded from the study. Consenting patients were subsequently recruited into the study and administered the study questionnaire which elicited patients’ demographics, household, environmental characteristics, associated symptoms and relevant background history. This included occupation, alcohol intake, dietary preferences and herbal remedies. Household structure was defined as follows: detached (a single house in an enclosed compound); semi-detached (two houses built side by side and sharing a common wall); compound structure (more than two separate houses enclosed in the same compound). Urban residences were categorized into class 1 – 3 based on the Accra Metropolitan Assembly (AMA) classification of Accra with tax imposition rates of Ghana Cedi (GHC) 100, GHC 60 and GHC 40 respectively. These reflect relative wealth and housing density in the Metropolis with class 1 corresponding to the most affluent and class 3 the most densely populated regions. Rural regions were geographical areas in Ghana not within an urban area and outside cities and towns.

*H. pylori* infection was confirmed by rapid-urease *Campylobacter*-like-organism (CLO) examination of antral biopsies at upper GI endoscopy (CLO testing kit: Cambridge Life Sciences Ltd, Cambridge, UK). Data was analyzed using the SPSS 16 Program. Categorical data was expressed as proportions and presented in tables. Chi-square was used to demonstrate the differences between observed variables with a p-value of <0.05 used to indicate statistical significance. Logistic regression analysis was used to demonstrate the relative risk of *H. pylori* infection in patients with specific predictor variables.

## Results

**Figure 1:**
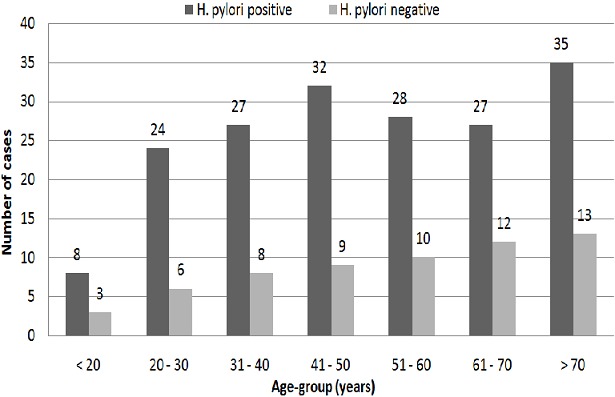
Age distribution of Helicobacter pylori cases

The sample population of dyspeptic patients attending the Endoscopy Unit for upper GI endoscopy yielded 242 patients of which 47.5% were females, 52.5% males. The overall prevalence of *H. pylori* obtained by immediate CLO-testing of gastric antral biopsies was 74.8%. The age distribution of *H. pylori-CLO*-positive cases was even across most age - groups as illustrated by Figure 1. Age group 21 – 30 years had the highest *H. pylori* prevalence, 80.0% (n = 24) while age group (61–70) had the lowest, 69.2% (n = 27). There was no statistically significant difference in *H. pylori* prevalence between age groups, (p = 0.957). Table 1 illustrates the relationship between housing characteristics and *H. pylori* prevalence including number in household, type of household and residential location. There was a statistically significant difference between residential classes in relation to *H. pylori* prevalence (p = 0.046) with rural areas having the highest prevalence (88.6%, n = 39). However, *H pylori* prevalence decreased across areas mapping to the three residential classes in accordance with increasing affluence (Table 1). Consistent with this theme, all nine patients with a household size over 30 individuals had *H. pylori* infection in comparison to 67.9%, (n = 55) of patients living in households with 1 – 5 members. By contrast, there was no statistical difference in *H. pylori* prevalence between household structural types; detached, semi-detached or compound house-structure, (p = 0.469).

**Table 1:** Housing characteristics and *Helicobacter pylori* prevalence

**Number of persons in household**	***Helicobacter pylori* cases**		**(n)**	**p-value**
	**Positive**	**Negative**		
1 – 5	55 (67.9%)	26 (32.1%)	81	*p* = 0.136
6 – 10	64 (81.0%)	15 (19.0%)	79	
11 – 20	33 (73.3%)	12 (26.7%)	45	
21 – 30	15 (78.9%)	4 (21.1%)	19	
> 30	9 (100%)	0 (0.0%)	9	
Unspecified	5 (55.5%)	4 (44.4%)	9	
Total	181 (74.8%)	61 (25.2%)	242	
*Household type	***Helicobacter pylori* cases**		**(n)**	**p-value**
	**Positive**	**Negative**		
Detached	72 (75.0%)	24 (25.0%)	96	*p* = 0.469
Semi-Detached	25 (80.6%)	6 (19.4%)	31	
Compound	81 (74.3%)	28 (25.7%)	109	
Unspecified	3 (50.0%)	3 (50.0%)	6	
Total	181 (74.8%)	61 (25.2%)	242	
**†Residential Class**	***Helicobacter pylori* cases**		**(n)**	**p-value**
	**Positive**	**Negative**		
Class 1	37 (64.9%)	20 (35.1%)	57	*p* = 0.046
Class 2	56 (71.8%)	22 (28.2%)	78	
Class 3	48 (77.4%)	14 (22.6%)	62	
Rural	39 (88.6%)	5 (11.4%)	44	
Total	180 (74.7%)	61 (25.3%)	241	

* Household structure defined detached (a single house in an enclosed compound); semi-detached (two houses built side by side and sharing a common wall); compound structure (more than two separate houses enclosed in the same compound)

† Urban residences categorized into class 1 – 3 based on the Accra Metropolitan Assembly (AMA) classification of Accra with tax imposition rates of Ghana Cedi (GHC) 100, GHC 60 and GHC 40 respectively

Table 2 shows the distribution of *H. pylori* cases by occupation; p = 0.107; the unemployed and patients involved in farming/agriculture had relatively high *H. pylori* infection rates of 92.3% and 91.7% respectively. Potential associations were also sought between consumption of alcohol, herbal remedies, dietary preference (spice, fat, smoked fish, salt) and *H. pylori* infection prevalence. However, none achieved statistical significance, Table 3. Table 4 demonstrates the relative risk of *H. pylori* infection with specific predictor variables after logistic regression analysis. Residential class was the most significant risk factor for the presence of *H. pylori* infection following multi-variate analysis (OR 1.86; 95% CI 1.27-2.73). Household size (OR 1.78; 95% CI 0.71-4.34) and structure (OR 2.37; 95% CI 0.92-6.09) were however potential risk factors for *H. pylori* infection.

**Table 2:** Distribution of *Helicobacter pylori* cases by occupation

Occupation	*Helicobacter pylori* cases		Total
	Positive	Negative	
Professional/ managerial	31 (68.9%)	14 (31.1%)	45
Sales/ services	54 (79.4%)	14 (20.6%)	68
Skilled manual	21 (61.8%)	13 (38.2%)	34
Unskilled manual	5 (71.4%)	2 (28.6%)	7
Farming	11 (91.7%)	1 (8.3%)	12
Unemployed	24 (92.3%)	2 (7.7%)	26
Retired	27 (67.5%)	13 (32.5%)	40
Other	8 (80.0%)	2 (20.0%)	10
Total	181 (74.6%)	61 (25.4%)	242

The relationship between occupation and prevalence of *H. pylori* infection

**Table 3:** Dietary preference, alcohol consumption and *Helicobacter pylori* infection

Dietary preference	*Helicobacter pylori* cases		Total	*p*-value
	Positive	Negative		
Salty	95 (76.6%)	29 (23.4%)	124	0.796
Spicy	105 (75.0%)	35 (25.0%)	140	0.585
Fatty	70 (78.7%)	19 (21.3%)	89	0.532
Smoked fish	159 (75.7%)	51 (24.3%)	210	0.297
Herbal medication use	94 (77.7%)	27 (22.3%)	121	0.300
Alcohol intake	45 (72.6%)	17 (27.4%)	62	0.636

The relationship between dietary preference, herbal preparation-use, alcohol intake and prevalence of *H. pylori* infection

**Table 4:** Predictor variables and *H. pylori* risk – multi-variate analysis

Predictor variable	Odds ratio	95% Confidence Interval
Age group	0.572	0.196 - 1.672
Gender	0.736	0.339 – 1.601
Number in household	1.757	0.712 – 4.337
Type of household	2.366	0.920 – 6.089
**Residential class**	**1.860**	**1.267 – 2.730**
Occupation	1.009	0.866 – 1.175
Smoker	0.211	0.008 – 5.399
Alcohol intake	1.096	0.362 – 3.318
Herbal medication use	1.182	0.567 – 2.465

Logistic regression analysis to demonstrate relative risk of *H. pylori* infection with specific predictor variables

## Discussion

The prevalence of *H. pylori* infection in this study was 74.8% comparable to previous studies in Ghana [7], Nigeria [10] and other developing countries [8]. The uniformly high age-related *H. pylori* prevalence demonstrated supports the notion that the rate of acquisition of infection is predominantly highest in early life [11]. Indeed, it is widely believed that once acquired colonization persists throughout life unless otherwise eradicated [11]. Re-infection with *H. pylori* following successful bacterial cure is not uncommon in developing countries, occurring in approximately ∼ 12% of individuals shown to have been originally cleared of this bacterium. By contrast, in developed countries∼1% such individuals are subsequently re-infected. Additionally, when re-infection occurs this has most commonly been shown to represent recrudescence of the original bacterial strain [12]. Studies based in Peru and Mexico showed re-infection rates of 3 – 7% [13, 14], while in comparison a European-based study elicited a rate of only 0.4% [15]. Re-infection from multiple sources of infection in developing countries, particularly in endemic areas has also been suggested as important in maintaining the presence of this organism during adulthood [8, 13, 14].

Rising numbers of household membership and household structure were not significantly associated with *H. pylori* infection. However, all nine patients with a household size of over 30 members had *H. pylori* infection in comparison to 67.9% of patients living in households with only 1 – 5 individuals. A study has shown that in UK adults, aged 50-- 59 years, sibling number was independently associated with prevalence of *H. pylori* infection [16]. Similarly, in a Cambodian study the number of children in the household was more significant than number of adults in predicting the presence of *H. pylori infection* [17, 18]. Specific characteristics of children may enhance the spread of *H. pylori* among contacts and household members. For example, a child may have an enhanced susceptibility to *H. pylori* through increased exposure and a possible lowered immune response [17].

In this study, occupation was used as a surrogate marker of socio-economic status of patients. Those who were classified as unemployed or students had a relatively high infection prevalence of 96.2%. Given the frequent correlation between employment status and wealth, these data would appear to be consistent with the finding that children from low-income families have significantly higher infection rates than those from high-income families [19]. The *H. pylori* prevalence in Black and Hispanic people was also inversely related to the social class during childhood. In support of the notion that childhood environment was critical, this relationship remained even after adjusting for the present social class and age of study participants [20]. More affluent and less densely populated urban areas (residential classes) also demonstrated lower infection rates (Table 1). Smaller family size, less crowding, improved sanitation and clean water help explain the decline in *H. pylori* prevalence in developed countries [17] as well as some developing countries [21]. This may be as a result of the reported presence of *H. pylori* in vomitus, human faeces and unclean water, all potentially serving as sources of infection of vulnerable residents in over-crowded and densely populated accommodations [16].

Farming and agriculture was associated with an infection prevalence of 91.7% suggesting possible zoonotic transmission. In Ghana, poultry, cows, sheep and goats are some of the common animals frequently reared in farming communities. Many animals such as cats, monkeys have been successfully infected with *H. pylori* strains and an increased risk of infection with exposure to sheep has also been reported in a number of studies [18, 22]. Since other *Helicobacter* species also strongly produce urease, the CLO test would not be able to differentiate *H. Pylori* from other species such as *H. Heilmannii* [23]. The large spiral gastric *Helicobacter-like* organisms (GHLOs), commonly noted in dogs and cats, often infect patients who own pets suggesting a zoonotic link. However, they are associated with approximately 0.08-1% of gastritis in humans [24]. Laboratory studies have experienced difficulty in isolating *H. Pylori* from material other than gastric tissue which has made identification of portals of entry and exit problematic [25]. Reports using whole-cell enzyme-linked immune-absorbent assay (ELISA) sonicate to monitor infection serologically, have cited a high incidence of *H. pylori* in abattoir workers [24]. These results have been thought to be confounded by potential antigenic cross-reactivity in workers′ sera due to the constant exposure to other gastrointestinal flora of animals [24]. Thus, the isolation of *H. pylori* from the inflamed gastric lining of commercially reared animals [26], and the ability to experimentally infect cats with *H. pylori* [24], continues to raise the possibility of zoonotic *H. pylori* transmission from infected animals who have close human contact.

## Conclusion

*H. pylori* is endemic in Ghana but the persistently high prevalence across age groups despite significant community anti-microbial use and abuse [27], suggests likely re-crudescence or re-infection from multiple sources in a developing country. Socio-cultural factors such as residential class and farming may be facilitating factors for its continued prevalence. Further study will evaluate the impact of these epidemiological factors, broader host traits and microbial determinants in the transmission and persistence of *H. pylori* in Ghana.

## References

[1] McGee DJ, May CA, Garner RM, Himpsl JM, Mobley HL (1999). Isolation of Helicobacter pylori genes that modulate urease activity. J Bacteriol..

[2] Cave DR (1996). Transmission and epidemiology of Helicobacter pylori. Am J Med..

[3] Pounder RE, Ng D (1995). The prevalence of Helicobacter pylori infection in different countries. Alimentary Pharmacology & Therapeutics..

[4] Parsonnet J, Friedman GD, Vandersteen DP, Chang Y, Vogelman JH, Orentreich N, Sibley RK (1991). Helicobacter pylori infection and the risk of gastric carcinoma. The New England Journal of Medicine..

[5] Bardhan PK (1997). Epidemiological features of Helicobacter pylori infection in developing countries. Clin Infect Dis..

[6] Segal I, Ally R, Mitchell H (2001). Helicobacter pylori--an African perspective. QJM..

[7] Baako BN, Darko R (1996). Incidence of Helicobacter pylori infection in Ghanaian patients with dyspeptic symptoms referred for upper gastrointestinal endoscopy. West African Journal of Medicine..

[8] Perez-Perez GI, Rothenbacher D, Brenner H (2004). Epidemiology of Helicobacter pylori infection. Helicobacter..

[9] Fox JG, Dangler CA, Taylor NS, King A, Koh TJ, Wang TC (1999). High-salt diet induces gastric epithelial hyperplasia and parietal cell loss, and enhances Helicobacter pylori colonization in C57BL/6 mice. Cancer Res..

[10] Jemilohun AC, Otegbayo JA, Ola SO, Oluwasola OA, Akere A (2010). Prevalence of Helicobacter pylori among Nigerian patients with dyspepsia in Ibadan. Pan Afr Med J..

[11] Rowland M, Daly L, Vaughan M, Higgins A, Bourke B, Drumm B (2006). Age-specific incidence of Helicobacter pylori. Gastroenterology..

[12] Morgan DR, Torres J, Sexton R, Herrero R, Salazar-Martinez E, Greenberg ER, Bravo LE, Dominguez RL, Ferreccio C, Lazcano-Ponce EC (2013). Risk of recurrent Helicobacter pylori infection 1 year after initial eradication therapy in 7 Latin American communities. JAMA..

[13] Soto G, Bautista CT, Roth DE, Gilman RH, Velapatino B, Ogura M, Dailide G, Razuri M, Meza R, Katz U (2003). Helicobacter pylori reinfection is common in Peruvian adults after antibiotic eradication therapy. J Infect Dis..

[14] Leal-Herrera Y, Torres J, Monath TP, Ramos I, Gomez A, Madrazo-de la Garza A, Dehesa-Violante M, Munoz O (2003). High rates of recurrence and of transient reinfections of Helicobacter pylori in a population with high prevalence of infection. Am J Gastroenterol..

[15] Cameron EA, Bell GD, Baldwin L, Powell KU, Williams SG (2006). Long-term study of re-infection following successful eradication of Helicobacter pylori infection. Aliment Pharmacol Ther..

[16] Ford AC, Forman D, Bailey AG, Goodman KJ, Axon AT, Moayyedi P (2007). Effect of sibling number in the household and birth order on prevalence of Helicobacter pylori: a cross-sectional study. Int J Epidemiol..

[17] Go MF (2002). Review article: natural history and epidemiology of Helicobacter pylori infection. Aliment Pharmacol Ther..

[18] Goodman KJ, Correa P, Tengana Aux HJ, Ramirez H, DeLany JP, Guerrero Pepinosa O, Lopez Quinones M, Collazos Parra T (1996). Helicobacter pylori infection in the Colombian Andes: a population-based study of transmission pathways. Am J Epidemiol..

[19] Klein PD, Graham DY, Gaillour A, Opekun AR, Smith EO (1991). Water source as risk factor for Helicobacter pylori infection in Peruvian children Gastrointestinal Physiology Working Group. Lancet..

[20] Malaty HM, Graham DY (1994). Importance of childhood socioeconomic status on the current prevalence of Helicobacter pylori infection. Gut..

[21] Parente JM, da Silva BB, Palha-Dias MP, Zaterka S, Nishimura NF, Zeitune JM (2006). Helicobacter pylori infection in children of low and high socioeconomic status in northeastern Brazil. Am J Trop Med Hyg..

[22] Dore MP, Sepulveda AR, El-Zimaity H, Yamaoka Y, Osato MS, Mototsugu K, Nieddu AM, Realdi G, Graham DY (2001). Isolation of Helicobacter pylori from sheep-implications for transmission to humans. Am J Gastroenterol..

[23] Solnick JV (2003). Clinical significance of Helicobacter species other than Helicobacter pylori. Clinical Infectious Diseases..

[24] Fox JG (1995). Non-human reservoirs of Helicobacter pylori. Alimentary Pharmacology & Therapeutics..

[25] Goodman KJ, Correa P (1995). The transmission of Helicobacter pylori. A critical review of the evidence. International Journal of Epidemiology..

[26] Handt LK, Fox JG, Dewhirst FE, Fraser GJ, Paster BJ, Yan LL, Rozmiarek H, Rufo R, Stalis IH (1994). Helicobacter pylori isolated from the domestic cat: public health implications. Infection and Immunity..

[27] Tagoe DNA (2009). A Study of Antibiotic Use and Abuse in Ghana: a case study of the Cape Coast Metropolis. The Internet Journal of Health..

